# 

**Published:** 2022-01

**Authors:** Ali Davoudi-Kiakalayeh

## Abstract

Dear Colleagues,

Although the drowning mortality rate has decreased in Iran from ten years ago, it is still considered a serious health problem. Guilan University of Medical Sciences is hosting the 4th National Conference on Drowning Prevention from 7th to 8th of September 2021 which is the fourth one in this series. We once again present a conference about the drowning prevention challenge in Iran. In addition, the event focuses on the following topics: drowning surveillance, national drowning registry system, and how to best utilize primary care system for implementation of drowning prevention strategies. We are also pleased to announce that the accepted abstracts will be published in the Journal of Injury and Violence Research (JIVR).

The Drowning Prevention Conference 2021 has been made possible with the support of Non- Communicable Disease Department, Deputy of Health, Ministry of Health and Medical Education, Iran, Karolinska Institutet, Sweden, and JIVR Team in Kermanshah University of Medical Sciences, Iran. I would like to offer my sincere thanks to all of the supporters especially Dr. Alireza Moghisi, head of Injury Department of MOH, Iran, Dr. Reza Mohammadi in Karolinska Institutet, Dr. Alireza Ahmadi in Kermanshah University of Medical Sciences, and Dr. Shahrokh Yousefzadeh-Chabok, chair of GRTRC, Iran.

Finally, I would like to extend my gratitude to the chancellor of Guilan University of Medical Sciences, Dr.Arsalan Salari for giving us the opportunity to hold this event.

